# Accidents and Emergencies

**Published:** 1881-01

**Authors:** 


### ACCIDENTS AND EMERGENCIES.

DR. HALL'S Family Doctor, or "Health at Home," is one of the most valuable books that a family can possess. We quote the following from the first three pages of the book :

The sting of a wasp, or bee, or yellow-jacket, has often proved fatal in five minutes, when the prompt application of hartshorn to the wound, and a few drops swallowed with water, would have antagonized the poison and saved the life ; but in a dozen houses in the country there might not be found a drop ; look under the head of "Bites and Stings," and it will be seen that a bit of soap or a handful of wood ashes stirred in a glass of water makes a hartshorn substitute in half a minute ; or, if there be not a wood fire in a mile, a handful of moist earth grabbed from the first mud-puddle, or pond, or brooklet's edge, contains hartshorn and other curative elements, which, if applied in the shape of a poultice gives instant relief to the sufferer.

Toward midnight, after the first sleep, the hateful croup usually fixes its dreadful fangs on the unconscious child. What avails it in the country, miles away from a physician or a drug store, that this, that, or the other remedy is "good for" the disease, when neither physician nor remedy could be had for hours ; and all this while the mother is in agony, and the infant sufferer clutches its throat for breath ? But turn to the proper heading in the book, and it will be seen that no medicine known is so potent for cure as a boiling tea-kettle and a bit of flannel ; or, as a lump of ice or snow, with a handful of salt, applied to the throat in a silken pad or bag.

The terrible cramp-colic, so often fatal before the dawn, can be relieved within an hour with a milk emetic, and flannels, wrung in boiling water, applied to the stomach.

Has a child, or other member of the household, swallowed a rank poison ? Turn to the article, and it will be seen in an instant that the number of poisons and their antidotes are legion ; yet all are comprised in two divisions—those which cause no pain whatever, and those which occasion fearful sufferings in the throat—that in both cases there are two things to be done : to dilute the poison, and get it out of the stomach the earliest moment possible ; the painful kind is to be met with drinking warm milk, or tepid, or even cold water, until the stomach can hold no more ; then a feather or finger in the throat will cause instant and copious vomiting. If there is no pain, then a more speedy method is to stir a tablespoonful each of salt and mustard in half a glass of water; the instant it reaches the stomach it begins to return, bringing everything else with it; and in either case, lest some of the poison might be left, go to the hen's nest, take a fresh egg or two, or more, and swallow the whites; or, if there is not a cow or hen in fifty miles, do without them; use warm water for the milk, and a tablespoonful of flour, stirred in half a glass of water, and drink down rapidly. It is a good substitute for the albumen of the egg.

It is in such ways that an effort is made to instruct the people how to avail themselves of nature's remedies for the cure of disease when neither medicines nor physicians are possible of securement; as in the accidents and burnings and maimings in connection with railroad traveling and disasters at sea.

The truth is, with some little understanding of the nature of disease, and some little genius for shifts and devices, half the ordinary ailments of humanity, in cases where at all curable by any human instrumentalities, can be successfully treated on a rock in the sea, on an iceberg at the poles, if there is fire, fresh air and water. The aim in all cases is to set before the reader certain cardinal points.

First. The nature of the disease.

Second. The remedy proposed.

Third. How the remedy accomplishes the object, that the patient may know whether the means used are doing what is wanted by the manifestations they are making, and if not, he may turn his attention to some other remedy acting in like manner.

There is a single idea on "Congestion" which will at once clear from the mind at least half of the mystery of medicine, and show how it is possible for one remedy to cure a hundred diseases. A house may be set on fire in many different ways. The effect of the fire is one, destruction; the remedy is one, water. Congestion may be brought about in a hundred different ways, in a hundred different parts of the body, giving rise to a hundred different diseases or symptoms; but the cause is one, congestion. The one thing to be done is the removal of that congestion; whatever does that will cure, if applied in time, whether it be by general hygienic measures, or by pure air, cleanliness, exercise and rest, or by hydropathic means, or the surer and more direct allopathic instrumentalities, as, for example, by the use of the "Liver

Pill" which all are instructed how to make, and which is destined to save multitudes.

The uninformed reader will be surprised to learn the curative virtues of some of the most familiar things in any common household, and surely it is the duty of all, especially of those who live in out-of-the-way places, to acquaint themselves with at least some of these, for in many instances it would be the saving of human life.

Take, for example, the varied uses of a good article of hog's lard. Two or three folds of woollen flannel dipped in hot hog's lard will most promptly remove the pain or swelling from the sting of hornet, wasp or bee.

2.—Warm, fresh, unsalted hog's lard is one of the very best things to dislodge any live insect from the ear

3.—A quarter of a pint of warmed lard drunk every fifteen minutes before breakfast, will sometimes dislodge a tape worm, head and all.

4.—Hog's lard, rubbed well into a swollen limb, with the hand patiently, often removes the swellings and any deep-seated pains attending them.

5.—Half a pint of hog's lard, taken on three successive mornings, has relieved cases of the most obstinate constipation, even when croton oil and injections had failed.

6.—If the skin, in scarlatina, is daily well rubbed with hog's lard, or bacon rind for ten days, it will allay the heat of the skin, remove the soreness of the throat, lessen the risk of dropsy, and prevent the spreading of the disease, by confining to the body those small solid particles, which, otherwise escaping from the skin, would be breathed into the lungs of others and swallowed into their stomachs, and thus infect the blood.

7.—The intolerable itching, which attends erysipelas is mitigated and entirely controlled by persistent inunctions with hog's lard.

8.—Persons who work in woollen factories, where the material has to receive hog's lard on it before it can be worked up, are remarkably exempt from consumptive disease, while those who live in cotton factories are especially liable to it.

9.—Inunctions daily and abundantly of the parts of the skin infected with itch will cure almost any ordinary case.

10.—The night sweats of consumption are often modified and sometimes removed by rubbing hog's lard into the skin every night, if sleeping in the same woollen night shirt, which becomes impregnated with the oil.

11.—There is no remedy which affords more instantaneous relief from the effect of swallowing acrid poisons than drinking hog's lard, which not only soothes the scalding heat in the throat, but dilutes the poison in the stomach, and, by continuing to drink it until the stomach is full, a feather in the throat will bring it all up, poison and all. Surely it is well to know these things, and any book must be valuable, a main subject of which is to utilize things which are most commonly at hand, with a view to alleviate pain, restore health, and save life.

A young man in a farmer's family accidentally dropped an open penknife, which struck the arm of a little girl playing on the floor. The blood flowed alarmingly fast; the father mounted a horse and rode at full speed for the nearest physician, who lived five miles away, to find that he had started an hour before to go ten miles in an opposite direction to visit a patient who was in a dangerous condition, and was not expected to return until next morning. A message was left, and in the most dreadful apprehension and suspense the father turned his face homeward. Meanwhile, the little child had bled to death; the mother was thrown into convulsions and expired. On learning the facts as he entered the house at nightfall, the father went through into the garden, threw himself into a well, and was drowned.

If a common handkerchief had been tied loosely about the arm, above the wounded artery, a stitch run through between the handkerchief and skin, and twisted around tightly, the bleeding would have been stopped in sixty seconds, and the child saved; but nobody in the house knew this.

It is to provide families and individuals with such knowledge as will enable them to act efficiently under the emergencies of sickness or accident that this book is written.

---

The manufacture of glucose from rags, the novel industry recently started in Germany, is regarded with much disfavor, and it is understood that the German Government will be likely to interfere with the business. The glucose is said to be chemically identical with grape sugar. The process, which is represented to be very cheap, is as follows: Old linen rags, which are composed of hard vegetable fibres, are converted into dextrine by the application of sulphuric acid, and the product thus obtained is then washed with milk of lime. Next it is treated with a stronger solution of the sulphuric acid than that first used, when the material is immediately transformed and crystalized into a glucose, from which appetizing jellies and tempting confections can be made.

